# An infectious SARS-CoV-2 B.1.1.529 Omicron virus escapes neutralization by therapeutic monoclonal antibodies

**DOI:** 10.21203/rs.3.rs-1175516/v1

**Published:** 2021-12-27

**Authors:** Laura VanBlargan, John Errico, Peter Halfmann, Seth Zost, James Crowe, Lisa Purcell, Yoshihiro Kawaoka, Davide Corti, Daved Fremont, Michael Diamond

**Affiliations:** Department of Medicine, Washington University School of Medicine; Department of Pathology & Immunology, Washington University School of Medicine; Influenza Research Institute, Department of Pathobiological Sciences, School of Veterinary Medicine, University of Wisconsin-Madison; Vanderbilt Vaccine Center, Vanderbilt University Medical Center; Department of Pediatrics Vanderbilt University Medical Center; Vanderbilt Vaccine Center, Vanderbilt University Medical Center; Department of Pediatrics Vanderbilt University Medical Center; Department of Pathology, and Microbiology and Immunology, Vanderbilt University Medical Center; Vir Biotechnology; Influenza Research Institute, Department of Pathobiological Sciences, School of Veterinary Medicine, University of Wisconsin-Madison; Division of Virology, Department of Microbiology and Immunology, Institute of Medical Science, University of Tokyo; The Research Center for Global Viral Diseases, National Center for Global Health and Medicine Research Institute, Tokyo; Humabs BioMed SA, a subsidiary of Vir Biotechnology; Department of Pathology & Immunology, Washington University School of Medicine; Department of Molecular Microbiology, Washington University School of Medicine; Department of Biochemistry & Molecular Biophysics, Washington University School of Medicine; Department of Medicine and Department of Pathology & Immunology and Department of Molecular Microbiology, Washington University School of Medicine; Andrew M. and Jane M. Bursky Center for Human Immunology and Immunotherapy Programs, Washington University School of Medicine; Center for Vaccines and Immunity to Microbial Pathogens, Washington University School of Medicine

**Keywords:** SARS-CoV-2, Omicron, therapeutic monoclonal antibodies

## Abstract

The emergence of the highly-transmissible B.1.1.529 Omicron variant of Severe acute respiratory syndrome coronavirus 2 (SARS-CoV-2) is concerning for antibody countermeasure efficacy because of the number of mutations in the spike protein. Here, we tested a panel of anti-receptor binding domain monoclonal antibodies (mAbs) corresponding to those in clinical use by Vir Biotechnology (S309, the parent mAb of VIR-7831 [Sotrovimab]), AstraZeneca (COV2–2196 and COV2–2130, the parent mAbs of AZD8895 and AZD1061), Regeneron (REGN10933 and REGN10987), Lilly (LY-CoV555 and LY-CoV016), and Celltrion (CT-P59) for their ability to neutralize an infectious B.1.1.529 Omicron isolate. Several mAbs (LY-CoV555, LY-CoV016, REGN10933, REGN10987, and CT-P59) completely lost neutralizing activity against B.1.1.529 virus in both Vero-TMPRSS2 and Vero-hACE2-TMPRSS2 cells, whereas others were reduced (COV2–2196 and COV2–2130 combination, ~12-fold decrease) or minimally affected (S309). Our results suggest that several, but not all, of the antibodies in clinical use may lose efficacy against the B.1.1.529 Omicron variant.

## Introduction

Since December of 2019, the global COVID-19 pandemic caused by SARS-CoV-2 has resulted in 267 million infections and 5.3 million deaths. The expansion of the COVID-19 pandemic and its accompanying morbidity, mortality, and destabilizing socioeconomic effects have made the development and distribution of SARS-CoV-2 therapeutics and vaccines an urgent global health priority^1^. While the rapid deployment of countermeasures including monoclonal antibodies and multiple highly effective vaccines has provided hope for curtailing disease and ending the pandemic, this has been jeopardized by emergence of more transmissible variants with mutations in the spike protein that also could evade protective immune responses.

Indeed, over the past year, several variant strains have emerged including B.1.1.7 (Alpha), B.1.351 (Beta), B.1.1.28 [also called P.1, Gamma]), and B.1.617.2 (Delta), among others, each having varying numbers of substitutions in the N-terminal domain (NTD) and the RBD of the SARS-CoV-2 spike. Cell-based assays with pseudoviruses or authentic SARS-CoV-2 strains suggest that neutralization by many EUA mAbs might be diminished against some of these variants, especially those containing mutations at positions L452, K477, and E484^2–6^. Notwithstanding this, *in vivo* studies in animals showed that when most EUA mAbs were used in combination they retained efficacy against different variants^7^. The recent emergence of B.1.1.529, the Omicron variant^8,9^, which has a larger number of mutations (~30 substitutions, deletions, or insertions) in the spike protein, has raised concerns that this variant will escape from protection conferred by vaccines and therapeutic mAbs.

## Results

**Figure 1:**
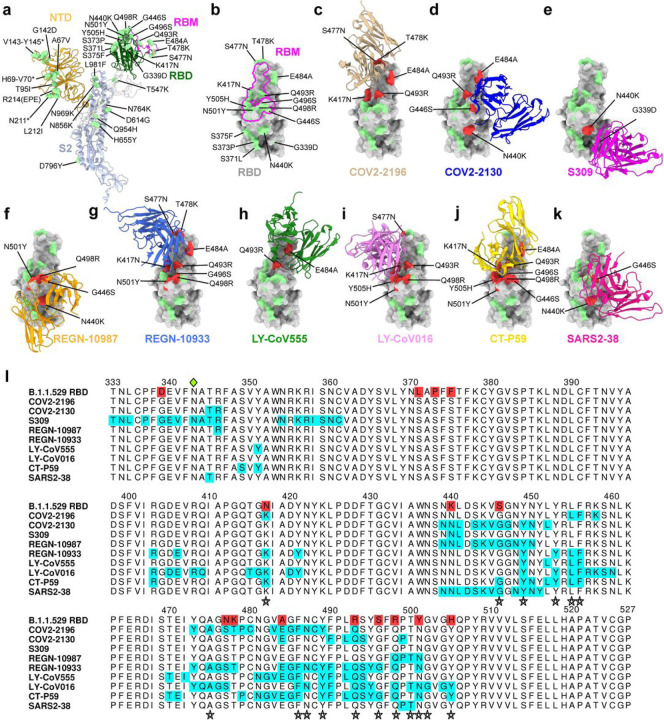
Neutralizing mAb epitopes on B.1.1.529. **a-b**, SARS-CoV-2 spike trimer (PDB: 7C2L and PDB: 6W41). One spike protomer is highlighted, showing the NTD in orange, RBD in green, RBM in magenta, and S2 portion of the molecule in blue (**a**). Close-up view of the RBD with the RBM outlined in magenta (**b**). Amino acids that are changed in B.1.1.529 compared to WA1/2020 are indicated in light green (**a-b**), with the exception of N679K and P681H, which were not modeled in the structures used. **c-k,** SARS-CoV-2 RBD bound by EUA mAbs COV2–2196 (**c**, PDB: 7L7D); COV2–2130 **(d**, PDB: 7L7E); S309 (**e**, PDB: 6WPS); REGN-10987 (**f**, PDB: 6XDG); REGN-10933 (**g**, PDB: 6XDG)); LY-CoV555 (**h**, PDB: 7KMG) LY-CoV016 (**i**, PDB: 7C01); CT-P59 (**j** PDB: 7CM4) and SARS2–38 (**k**, PDB: 7MKM). Residues mutated in the B.1.1.529 RBD and contained in these mAbs respective epitopes are shaded red, whereas those outside the epitope are shaded green. **l**, multiple sequence alignment showing the epitope footprints of each EUA mAb on the SARS-CoV-2 RBD highlighted in cyan. B.1.1.529 RBD is shown in the last row, with sequence changes relative to the WT RBD highlighted red. A green diamond indicates the location of the N-linked glycan at residue 343. Stars below the alignment indicate hACE2 contact residues on the SARS-CoV-2 RBD40.

We obtained an infectious clinical isolate of B.1.1.529 from a symptomatic individual in the United States (hCoV-19/USA/WI-WSLH-221686/2021). We propagated the virus once in Vero cells expressing transmembrane protease serine 2 (TMPRSS2) to prevent the emergence of adventitious mutations at or near the furin cleavage site in the spike protein^10^. Our B.1.1.529 isolate encodes the following mutations in the spike protein (A67V, D69–70, T95I, G142D, D143–145, D211, L212I, insertion 214EPE, G339D, S371L, S373P, S375F, K417N, N440K, G446S, S477N, T478K, E484A, Q493R, G496S, Q498R, N501Y, Y505H, T547K, D614G, H655Y, N679K, P681H, N764K, D796Y, N856K, Q954H, N969K, and L981F; Fig 1a–b and GISAID: EPI_ISL_7263803), which is similar to strains identified in Africa^11^. Our isolate, however, lacks an R346K mutation, which is present in a minority (~8%) of reported strains.

Given the number of substitution in the B.1.1.529 spike protein, including eight amino acid changes (K417N, G446S, S477N, Q493R, G496S, Q498R, N501Y, Y505H) in the ACE2 receptor binding motif (RBM), we first evaluated possible effects on the structurally-defined binding epitopes^12,13^ of mAbs corresponding to those with EUA approval or in advanced clinical development (S309 [parent of VIR-7831 (Sotrovimab), RBD group III]^14,15^; COV2–2196 (RBD group I) and COV2–2130 (RBD group III)[parent mAbs of AZD8895 and AZD1061, respectively]^16^; REGN10933 (RBD group I) and REGN10987 (RBD group III)^17^, LY-CoV555 (RBD group I) and LY-CoV016 (RBD group I)^18,19^; and CT-P59 [Celltrion, RBD group I]^20^) along with an additional broadly neutralizing mAb (SARS2–38, (RBD group II)) that we recently described^21^. We mapped the B.1.1.529 spike mutations onto the antibody-bound SARS-CoV-2 spike or RBD structures published in the RCSB Protein Data Bank (Fig 1c–k). While every antibody analyzed had structurally defined recognition sites that were altered in the B.1.1.529 spike, the differences varied among mAbs with some showing larger numbers of changed residues (Fig 1l: COV2–2196, n = 5; COV2–2130, n = 4; S309, n = 2; REGN10987, n = 4; REGN10933, n = 8; Ly-CoV555, n = 2; Ly-CoV016, n = 6; CT-P59, n = 8; and SARS2–38, n = 2).

**Figure 2:**
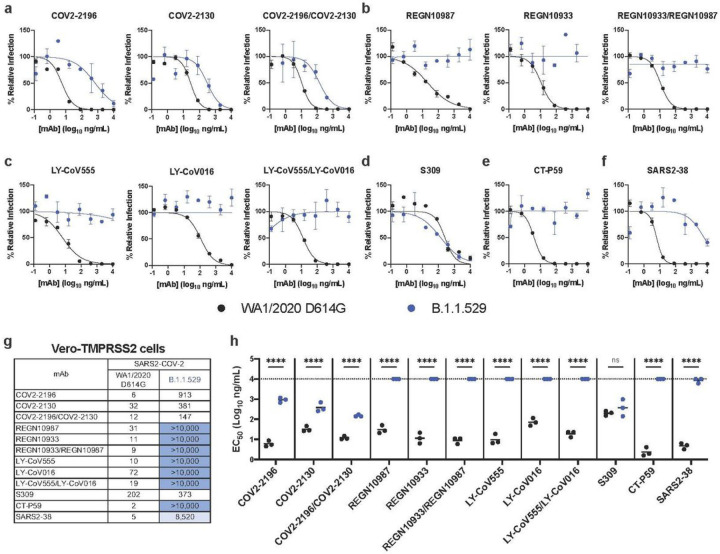
Neutralization of SARS-CoV-2 B.1.1.529 Omicron strain by mAbs in Vero-TMPRSS2 cells. **a-f,** Neutralization curves in Vero-TMPRSS2 cells comparing the sensitivity of SARS-CoV-2 strains with the indicated mAbs (COV2–2196, COV2–2130; REGN10933, REGN10987, LY-CoV555, LY-CoV016, S309, CT-P59, and SARS2–38) with WA1/2020 D614G and B.1.1.529. Also shown are the neutralization curves for antibody cocktails (COV2–2196/COV2–2130, REGN10933/REGN10987, or LY-CoV555/LY-CoV016). For data with mAb combinations, the x-axis represents the total concentration of mAb used. One representative experiment of three performed in technical duplicate is shown. Error bars indicate range of technical replicates. Data (% relative infection) are normalized to a no mAb control. **g**, Summary of EC_50_ values (ng/ml) of neutralization of SARS-CoV-2 viruses (WA1/2020 D614G and B.1.1.529) performed in Vero-TMPRSS2 cells. Data is the geometric mean of 3 experiments. Blue shading: light, EC_50_ > 5,000 ng/mL; dark, EC_50_ > 10,000 ng/mL. **h**, Comparison of EC_50_ values by mAbs against WA1/2020 D614G and B.1.1.529 (3 experiments, ns, not significant; ****, *P* < 0.0001; two-way ANOVA with Sidak’s post-test). Each symbol represents neutralization data from an individual experiment. Bars indicate mean values. The dotted line indicates the upper limit of dosing of the assay.

To address the functional significance of the spike sequence variation in B.1.1.529 for antibody neutralization, we used a high-throughput focus reduction neutralization test (FRNT)^22^ with WA1/2020 D614G and B.1.1.529 in Vero-TMPRSS2 cells (Fig 2). We tested individual and combinations of mAbs that target the RBD in Vero-TMPRSS2 cells including S309 (Vir Biotechnology), COV2–2130/COV2–2196 (parent mAbs of AZD1061 and AZD8895 provided by Vanderbilt University Medical Center), REGN10933/REGN10987 (synthesized based on casirivimab and imdevimab sequences from Regeneron), LY-CoV555/LY-CoV016 (synthesized based on bamlanivimab and etesevimab sequences from Lilly), CT-P59 (synthesized based on regdanvimab sequences from Celltrion), and SARS2–38. As expected, all individual or combinations of mAbs tested neutralized the WA1/2020 D614G isolate with EC_50_ values similar to published data^6,20,23^. However, when tested alone, REGN10933, REGN10987, LY-CoV555, LV-CoV016, CT-P59 and SARS2–38 completely lost neutralizing activity against B.1.1.529, with little inhibitory capacity even at the highest (10,000 ng/mL) concentration tested. COV2–2130 and COV2–2196 showed an intermediate ~12 to 150-fold (*P* < 0.0001) loss in inhibitory activity, respectively against the B.1.1.529 strain. In comparison, S309 showed a less than 2-fold (*P* > 0.5) reduction in neutralizing activity against B.1.1.529 (Fig 2a–h). Analysis of mAb combinations currently in clinical use showed that REGN10933/REGN10987 and LY-CoV555/LV-CoV016 lost all neutralizing activity against B.1.1.529, whereas COV2–2130/COV2–2196 showed a ~12-fold (*P* < 0.0001) reduction in inhibitory activity from an EC_50_ of 12 to 147 ng/mL.

**Figure 3:**
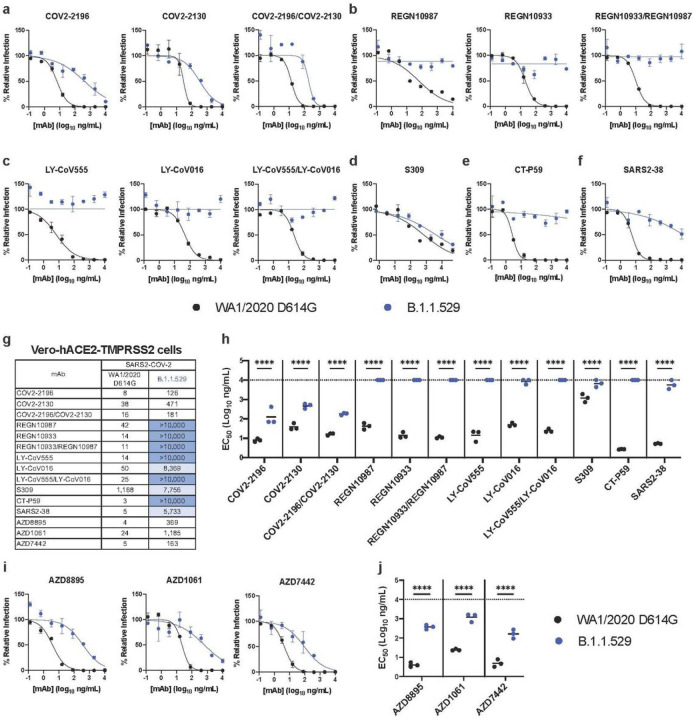
Neutralization of SARS-CoV-2 B.1.1.529 Omicron strain by mAbs in Vero-hACE2-TMPRSS2 cells. **a-f**, Neutralization curves in Vero-hACE2-TMPRSS2 cells comparing the sensitivity of SARS-CoV-2 strains with the indicated mAbs (S309, COV2–2196, COV2–2130; REGN10933, REGN10987, LY-CoV555, LY-CoV016, CT-P59, and SARS2–38) with WA1/2020 D614G and B.1.1.529. Also shown are the neutralization curves for antibody cocktails (COV2–2196/COV2–2130, REGN10933/REGN10987, or LY-CoV555/LY-CoV016). For data with mAb combinations, the x-axis represents the total concentration of mAb used. One representative experiment of three performed in technical duplicate is shown. Error bars indicate range of technical replicates. Data (% relative infection) are normalized to a no mAb control. **g**, Summary of EC_50_ values (ng/ml) of neutralization of SARS-CoV-2 viruses (WA1/2020 D614G and B.1.1.529) performed in Vero-hACE2-TMPRSS2 cells. Data is the geometric mean of 3 experiments. Blue shading: light, EC_50_ > 5,000 ng/mL; dark, EC_50_ > 10,000 ng/mL. **h**, Comparison of EC_50_ values by mAbs against WA1/2020 D614G and B.1.1.529. **i-j**, Neutralization curves in Vero-hACE2-TMPRSS2 cells comparing WA1/2020 D614G and B.1.1.529 infection in the presence of AZD1061, AZD8895, and the combination AZD7442. **h** and **j**. 3 experiments, ns, not significant; ****, *P* < 0.0001; two-way ANOVA with Sidak’s post-test. Each symbol represents neutralization data from an individual experiment. Bars indicate mean values. The dotted line indicates the upper limit of dosing of the assay.

We repeated experiments in Vero-hACE2-TMPRSS2 cells to account for effects of hACE2 expression, which can affect neutralization by some anti-SARS-CoV-2 mAbs^21,24^. Moreover, modeling studies suggest that the mutations in the B.1.1.529 spike may enhance interactions with hACE2^25^. All individual or combinations of mAbs tested neutralized the WA1/2020 D614G isolate as expected. However, REGN10933, REGN10987, LY-CoV555, LV-CoV016, SARS2–38, and CT-P59 completely lost neutralizing activity against B.1.1.529, and the combinations of REGN10933/ REGN10987 or LY-CoV555/LV-CoV016 also lacked inhibitory capacity (Fig 3a–h). In comparison, COV2–2130 and COV2–2196 showed reduced activity (~12 and 16-fold, respectively; *P* < 0.0001) against B.1.1.529 as did the combination of COV2–2130/COV2–2196 mAbs (~11-fold, *P* < 0.0001). The S309 mAb exhibited less potent neutralizing activity in Vero-hACE2-TMPRSS2 cells against WA1/2020 D614G virus with a flatter dose response curve (Fig 3d), as seen previously^6,26^, and showed a moderate (~6-fold, *P* < 0.0001) reduction in neutralizing activity against B.1.1.529. Thus, while the trends in mAb neutralization of B.1.1.529 generally were similar to Vero-TMPRSS2 cells, some minor differences in potency were noted in cells expressing hACE2.

## Discussion

Our experiments show a marked loss of inhibitory activity by several of the most highly neutralizing mAbs that are in advanced clinical development or have EUA approval. We evaluated antibodies that correspond to monotherapy or combination therapy that have shown pre- and post-exposure success in clinical trials and patients infected with historical SARS-CoV-2 isolates. Our results confirm *in silico* predictions of how amino acid changes in B.1.1.529 RBD might negatively impact neutralizing antibody interactions^18,27^. Moreover, they agree with preliminary studies showing that several clinically used antibodies lose neutralizing activity against B.1.1.529 spike-expressing recombinant lentiviral or vesicular stomatitis virus (VSV)-based pseudoviruses^28–30^. One difference is that our study with authentic B.1.1.529 showed only moderately reduced neutralization by antibodies corresponding to the AstraZeneca combination (COV2–2196 and COV2–2130); in contrast, another group reported escape of these mAbs using a VSV pseudovirus displaying a B.1.1.529 spike protein in Huh7 hepatoma cells^29^. Additional studies are needed to determine whether this disparity in results is due to the cell type, the virus (authentic versus pseudotype), or preparation and combination of antibody. To begin to address this issue, we obtained AZD1061, AZD8895, and the combination AZD7442 directly from the manufacturer and tested them for neutralization of WA1/2020 D614G and B.1.1529 in Vero-hACE2-TMPRSS2 cells. We observed relatively similar reductions in inhibitory activity compared to the preclinical COV2–2130 and COV2–2196 mAbs with 49, 92, and 33-fold lower EC_50_ values against B.1.1.529 by AZD1061, AZD8895, and AZD7442, respectively (Fig 3g, i, and j).

While the Regeneron (REGN10933 and REGN10987), Lilly (LY-CoV555 and LV-CoV016) and Celltrion (CT-P59) antibodies or combinations showed an almost complete loss of neutralizing activity against B.1.1.529, in our assays with Vero-TMPRSS2 and Vero-hACE2-TMPRSS2 cells, the mAbs corresponding to the AstraZeneca combination (COV-2196 and COV-2130) or Vir Biotechnology (S309) products retained substantial inhibitory activity. Although these data suggest that some of mAbs in clinical use may retain benefit, validation experiments *in vivo*^7^ are needed to support this conclusion and inform clinical decisions.

Given the loss of inhibitory activity against B.1.1.529 of many highly neutralizing anti-RBD mAbs in our study, it appears likely that serum polyclonal antibody responses generated after vaccination or natural infection also may lose substantial inhibitory activity against B.1.1.529, which could compromise protective immunity and explain a rise in symptomatic infections in vaccinated individuals^31^. Indeed, studies have reported approximately 25 to 40-fold reductions in serum neutralizing activity compared to historical D614G-containing strains from individuals immunized with the Pfizer BNT162b2 and AstraZeneca AZD1222 vaccines^28,30,32,33^.

We note several limitations of our study: (1) Our experiments focused on the impact of the extensive sequence changes in the B.1.1.529 spike protein on mAb neutralization in cell culture. Despite observing differences in neutralizing activity with certain mAbs, it remains to be determined how this finding translates into effects on clinical protection against B.1.1.529; (2) Although virus neutralization is a correlate of immune protection against SARS-CoV-2^7,34,35^, this measurement does not account for Fc effector functions if antibodies residually bind B.1.1.529 spike proteins on the virion or surface of infected cells. Fcg receptor or complement protein engagement by spike binding antibodies could confer substantial protection^36–38^; (3) We used the prevailing B.1.1.529 Omicron isolate that lacks an R346K mutation. While only 8.3% of B.1.1.529 sequences in GISAID (accessed on 12/14/2021) have an R346K mutation, this substitution might further affect neutralization of some of the clinically used mAbs given that R346 is a contact residue for COV2–2130, REGN10987, and S309 (Fig 1l). At least for S309, the R346K mutation did not impact neutralization of pseduoviruses displaying B.1.1.529 spike proteins^30^. Nonetheless, studies with infectious B.1.1.529 isolates with R346K mutations may be warranted if the substitution becomes more prevalent; (4) Our data is derived from experiments with Vero-TMPRSS2 and Vero-hACE2-TMPRSS2 cells. While these cells standardly are used to measure antibody neutralization of SARS-CoV-2 strains, primary cells targeted by SARS-CoV-2 *in vivo* can express unique sets of attachment and entry factors^39^, which could impact receptor and entry blockade by specific antibodies. Indeed, prior studies have reported that the cell line used can affect the potency of antibody neutralization against different SARS-CoV-2 variants^6^.

In summary, our cell culture-based analysis of neutralizing mAb activity against an authentic infectious B.1.1.529 Omicron SARS-CoV-2 isolate suggests that several, but not all, existing therapeutic antibodies will lose protective benefit. Thus, the continued identification and use of broadly and potently neutralizing mAbs that target the most highly conserved residues on the SARS-CoV-2 spike likely is needed to prevent resistance against B.1.1.529 and future variants with highly mutated spike sequences.

## Methods

### Cells.

Vero-TMPRSS2^41^ and Vero-hACE2-TMPRSS2^6^ cells were cultured at 37°C in Dulbecco’s Modified Eagle medium (DMEM) supplemented with 10% fetal bovine serum (FBS), 10 mM HEPES pH 7.3, and 100 U/ml of penicillin–streptomycin. Vero-TMPRSS2 cells were supplemented with 5 mg/mL of blasticidin. Vero-hACE2-TMPRSS2 cells were supplemented with 10 µg/mL of puromycin. All cells routinely tested negative for mycoplasma using a PCR-based assay.

### Viruses.

The WA1/2020 recombinant strain with substitutions (D614G) was described previously^42^. The B.1.1.529 isolate (hCoV-19/USA/WI-WSLH-221686/2021) was obtained from an individual in Wisconsin as a midturbinate nasal swab and passaged once on Vero-TMPRSS2 cells^43^. All viruses were subjected to next-generation sequencing (GISAID: EPI_ISL_7263803) to confirm the stability of substitutions. All virus experiments were performed in an approved biosafety level 3 (BSL-3) facility.

### Monoclonal antibody purification.

The mAbs used in this paper (COV2–2196, COV2–2130, S309, REGN10933, REGN10987, LY-CoV555, LY-CoV016, CT-P59, SARS2–38, AZD1061, AZD8895, and AZD7442) have been described previously^14,17,21,44–48^. S309 is the parent of VIR-7831 (Sotrovimab); the clinically used mAb is engineered for enhanced clinical developability, as reported previously^23^. COV2–2196 and COV2–2130 mAbs were produced after transient transfection using the Gibco ExpiCHO Expression System (ThermoFisher Scientific) following the manufacturer’s protocol. Culture supernatants were purified using HiTrap MabSelect SuRe columns (Cytiva, formerly GE Healthcare Life Sciences) on an AKTA Pure chromatographer (GE Healthcare Life Sciences). Purified mAbs were buffer-exchanged into PBS, concentrated using Amicon Ultra-4 50-kDa centrifugal filter units (Millipore Sigma) and stored at −80 °C until use. Purified mAbs were tested for endotoxin levels (found to be less than 30 EU per mg IgG). Endotoxin testing was performed using the PTS201F cartridge (Charles River), with a sensitivity range from 10 to 0.1 EU per mL, and an Endosafe Nexgen-MCS instrument (Charles River). S309, REGN10933, REGN10987, LY-CoV016, LY-CoV555, CT-P59, and SARS2–38 mAb proteins were produced in CHOEXPI or EXPI293F cells and affinity purified using HiTrap Protein A columns (GE Healthcare, HiTrap mAb select Xtra #28–4082-61). Purified mAbs were suspended into 20 mM histidine, 8% sucrose, pH 6.0 or PBS. The final products were sterilized by filtration through 0.22 μm filters and stored at 4°C.

### Focus reduction neutralization test.

Serial dilutions of mAbs were incubated with 10^2^ focus-forming units (FFU) of SARS-CoV-2 (WA1/2020 D614G or B.1.1.529) for 1 h at 37°C. Antibody-virus complexes were added to Vero-TMPRSS2 or Vero-hACE2-TMPRSS2 cell monolayers in 96-well plates and incubated at 37°C for 1 h. Subsequently, cells were overlaid with 1% (w/v) methylcellulose in MEM. Plates were harvested at 30 h (WA1/2020 D614G on Vero-TMPRSS2 cells), 70 h (B.1.1.529 on Vero-TMPRSS2 cells), or 24 h (both viruses on Vero-hACE2-TMPRSS2 cells) later by removal of overlays and fixation with 4% PFA in PBS for 20 min at room temperature. A longer time of incubation was required for B.1.1.529-infected Vero-TMPRSS2 cells because the foci were smaller at the time point and difficult to quantitate. Plates with WA1/2020 D614G were washed and sequentially incubated with an oligoclonal pool (1 mg/ml of each) of SARS2–2, SARS2–11, SARS2–16, SARS2–31, SARS2–38, SARS2–57, and SARS2–71^49^ anti-S antibodies. Plates with B.1.1.529 were additionally incubated with a pool of mAbs that cross-react with SARS-CoV-1 and bind a CR3022-competing epitope on the RBD^21^. All plates were subsequently stained with HRP-conjugated goat anti-mouse IgG (Sigma, A8924, 1:1,000) in PBS supplemented with 0.1% saponin and 0.1% bovine serum albumin. SARS-CoV-2-infected cell foci were visualized using TrueBlue peroxidase substrate (KPL) and quantitated on an ImmunoSpot microanalyzer (Cellular Technologies). Data (% relative infection) are normalized to a no antibody control. Antibody-dose response curves were analyzed using non-linear regression analysis with a variable slope (GraphPad Software), and the half-maximal inhibitory concentration (EC_50_) was calculated.

### Model of mAb-B.1.1.529 spike complexes.

The spike model is a composite of data from PDB: 7C2L and PDB: 6W41. Models of mAb complexes were generated from their respective PDB files with the following accession codes: COV2–2196 (PDB: 7L7D); COV2–2130 (PDB: 7L7E); S309 (PDB: 6WPS); REGN-10987 (PDB: 6XDG); REGN-10933 (PDB: 6XDG)); LY-CoV555 (PDB: 7KMG) LY-CoV016 (PDB: 7C01); CT-P59 (PDB: 7CM4) and SARS2–38 (PDB: 7MKM). Epitope footprints used in the multiple sequence alignment were determined using PISA interfacial analysis on the various mAb:RBD complexes^50^. Structural figures were generated using UCSF ChimeraX^51^.

### Data availability.

All data supporting the findings of this study are available within the paper, in the Source Data, and from the corresponding author upon request. There are no restrictions in obtaining access to primary data.

### Code availability.

No code was used in the course of the data acquisition or analysis.

### Reagent availability.

All reagents described in this paper are available through Material Transfer Agreements. AZD8895 and AZD1061 may be obtained from AstraZeneca for non-commercial internal research purposes under material transfer agreements upon request.

### Statistical analysis.

The number of independent experiments and technical replicates used are indicated in the relevant Figure legends. A two-way ANOVA with Sidak’s post-test was used for comparisons of antibody potency between WA1/2020 D614G and B.1.1.59.

## Supplementary Material

Supplement 1

Supplement 2

## References

[1] Sempowski G.D., Saunders K.O., Acharya P., Wiehe K.J. & Haynes B.F. Pandemic Preparedness: Developing Vaccines and Therapeutic Antibodies For COVID-19. Cell 181, 1458–1463 (2020). 32492407 10.1016/j.cell.2020.05.041 PMC7250787

[2] Wibmer C.K., SARS-CoV-2 501Y.V2 escapes neutralization by South African COVID-19 donor plasma. bioRxiv (2021). 10.1038/s41591-021-01285-x 33654292

[3] Wang Z., mRNA vaccine-elicited antibodies to SARS-CoV-2 and circulating variants. Nature (2021). 10.1038/s41586-021-03324-6 PMC8503938 33567448

[4] Tada T., Neutralization of viruses with European, South African, and United States SARS-CoV-2 variant spike proteins by convalescent sera and BNT162b2 mRNA vaccine-elicited antibodies. bioRxiv (2021).

[5] Wang P., Antibody Resistance of SARS-CoV-2 Variants B.1.351 and B.1.1.7. Nature (2021). 10.1038/s41586-021-03398-2 33684923

[6] Chen R.E., Resistance of SARS-CoV-2 variants to neutralization by monoclonal and serum-derived polyclonal antibodies. Nat Med (2021). 10.1038/s41591-021-01294-w PMC8058618 33664494

[7] Chen R.E., In vivo monoclonal antibody efficacy against SARS-CoV-2 variant strains. Nature (2021). 10.1038/s41586-021-03720-y PMC8349859 34153975

[8] Callaway E. & Ledford H. How bad is Omicron? What scientists know so far. Nature (2021). 10.1038/d41586-021-03614-z 34857948

[9] Torjesen I. Covid-19: Omicron may be more transmissible than other variants and partly resistant to existing vaccines, scientists fear. BMJ (Clinical research ed 375, n2943 (2021). 34845008 10.1136/bmj.n2943

[10] Johnson B.A., Loss of furin cleavage site attenuates SARS-CoV-2 pathogenesis. Nature (2021). 10.1038/s41586-021-03237-4 PMC8175039 33494095

[11] Chen J., Wang R., Gilby N.B. & Wei G.W. Omicron (B.1.1.529): Infectivity, vaccine breakthrough, and antibody resistance. ArXiv (2021). 10.1021/acs.jcim.1c01451 PMC8751645 34989238

[12] Barnes C.O., SARS-CoV-2 neutralizing antibody structures inform therapeutic strategies. Nature 588, 682–687 (2020). 33045718 10.1038/s41586-020-2852-1 PMC8092461

[13] Greaney A.J., Mapping mutations to the SARS-CoV-2 RBD that escape binding by different classes of antibodies. Nat Commun 12, 4196 (2021). 34234131 10.1038/s41467-021-24435-8 PMC8263750

[14] Pinto D., Cross-neutralization of SARS-CoV-2 by a human monoclonal SARS-CoV antibody. Nature 583, 290–295 (2020). 32422645 10.1038/s41586-020-2349-y

[15] Gupta A., Early Treatment for Covid-19 with SARS-CoV-2 Neutralizing Antibody Sotrovimab. N Engl J Med 385, 1941–1950 (2021). 34706189 10.1056/NEJMoa2107934

[16] Zost S.J., Potently neutralizing and protective human antibodies against SARS-CoV-2. Nature 584, 443–449 (2020). 32668443 10.1038/s41586-020-2548-6 PMC7584396

[17] Baum A., REGN-COV2 antibodies prevent and treat SARS-CoV-2 infection in rhesus macaques and hamsters. Science (2020). 10.1126/science.abe2402 PMC7857396 33037066

[18] Starr T.N., Greaney A.J., Dingens A.S. & Bloom J.D. Complete map of SARS-CoV-2 RBD mutations that escape the monoclonal antibody LY-CoV555 and its cocktail with LY-CoV016. Cell reports. Medicine, 100255 (2021). 33842902 10.1016/j.xcrm.2021.100255 PMC8020059

[19] Gottlieb R.L., Effect of Bamlanivimab as Monotherapy or in Combination With Etesevimab on Viral Load in Patients With Mild to Moderate COVID-19: A Randomized Clinical Trial. Jama 325, 632–644 (2021). 33475701 10.1001/jama.2021.0202 PMC7821080

[20] Kim C., A therapeutic neutralizing antibody targeting receptor binding domain of SARS-CoV-2 spike protein. Nat Commun 12, 288 (2021). 33436577 10.1038/s41467-020-20602-5 PMC7803729

[21] VanBlargan L.A., A potently neutralizing SARS-CoV-2 antibody inhibits variants of concern by utilizing unique binding residues in a highly conserved epitope. Immunity (2021). 10.1016/j.immuni.2021.08.016 PMC8373659 34481543

[22] Case J.B., Neutralizing antibody and soluble ACE2 inhibition of a replication-competent VSV-SARS-CoV-2 and a clinical isolate of SARS-CoV-2. Cell Host and Microbe 28, 475–485 (2020). 32735849 10.1016/j.chom.2020.06.021 PMC7332453

[23] Cathcart A.L., The dual function monoclonal antibodies VIR-7831 and VIR-7832 demonstrate potent in vitro and in vivo activity against SARS-CoV-2. bioRxiv, 2021.2003.2009.434607 (2021).

[24] Suryadevara N., Neutralizing and protective human monoclonal antibodies recognizing the N-terminal domain of the SARS-CoV-2 spike protein. Cell 184, 2316–2331.e2315 (2021). 33773105 10.1016/j.cell.2021.03.029 PMC7962591

[25] Golcuk M., Yildiz A. & Gur M. The Omicron Variant Increases the Interactions of SARS-CoV-2 Spike Glycoprotein with ACE2. bioRxiv, 2021.2012.2006.471377 (2021). 10.1016/j.jmgm.2022.108286 PMC9352197 35964366

[26] Lempp F.A., Lectins enhance SARS-CoV-2 infection and influence neutralizing antibodies. Nature 598, 342–347 (2021). 34464958 10.1038/s41586-021-03925-1

[27] Ford C.T., Machado D.J. & Janies D.A. Predictions of the SARS-CoV-2 Omicron Variant (B.1.1.529) Spike Protein Receptor-Binding Domain Structure and Neutralizing Antibody Interactions. bioRxiv, 2021.2012.2003.471024 (2021).

[28] Wilhelm A., Reduced Neutralization of SARS-CoV-2 Omicron Variant by Vaccine Sera and monoclonal antibodies. medRxiv : the preprint server for health sciences, 2021.2012.2007.21267432 (2021).

[29] Cao Y.R., B.1.1.529 escapes the majority of SARS-CoV-2 neutralizing antibodies of diverse epitopes. bioRxiv, 2021.2012.2007.470392 (2021).

[30] Cameroni E., Broadly neutralizing antibodies overcome SARS-CoV-2 Omicron antigenic shift. bioRxiv, 2021.2012.2012.472269 (2021). 10.1038/s41586-021-04386-2 PMC9531318 35016195

[31] Callaway E. Omicron likely to weaken COVID vaccine protection. Nature (2021). 10.1038/d41586-021-03672-3 34880488

[32] Cele S., SARS-CoV-2 Omicron has extensive but incomplete escape of Pfizer BNT162b2 elicited neutralization and requires ACE2 for infection. medRxiv : the preprint server for health sciences, 2021.2012.2008.21267417 (2021).

[33] Dejnirattisai W., Reduced neutralisation of SARS-COV-2 Omicron-B.1.1.529 variant by post-immunisation serum. medRxiv : the preprint server for health sciences, 2021.2012.2010.21267534 (2021). 10.1016/S0140-6736(21)02844-0 PMC8687667 34942101

[34] Kim J.H., Marks F. & Clemens J.D. Looking beyond COVID-19 vaccine phase 3 trials. Nat Med (2021). 10.1038/s41591-021-01230-y 33469205

[35] Khoury D.S., Neutralizing antibody levels are highly predictive of immune protection from symptomatic SARS-CoV-2 infection. Nat Med 27, 1205–1211 (2021). 34002089 10.1038/s41591-021-01377-8

[36] Schäfer A., Antibody potency, effector function, and combinations in protection and therapy for SARS-CoV-2 infection in vivo. J Exp Med 218(2021). 10.1084/jem.20201993 PMC7673958 33211088

[37] Zohar T., Compromised Humoral Functional Evolution Tracks with SARS-CoV-2 Mortality. Cell 183, 1508–1519.e1512 (2020). 33207184 10.1016/j.cell.2020.10.052 PMC7608014

[38] Winkler E.S., Human neutralizing antibodies against SARS-CoV-2 require intact Fc effector functions for optimal therapeutic protection. Cell 184, 1804–1820.e1816 (2021). 33691139 10.1016/j.cell.2021.02.026 PMC7879018

[39] Bailey A.L. & Diamond M.S. A Crisp(r) New Perspective on SARS-CoV-2 Biology. Cell 184, 15–17 (2021). 33338422 10.1016/j.cell.2020.12.003 PMC7746090

[40] Lan J., Structure of the SARS-CoV-2 spike receptor-binding domain bound to the ACE2 receptor. Nature 581, 215–220 (2020). 32225176 10.1038/s41586-020-2180-5

